# Aging phenomics enabled by quantitative imaging analysis

**DOI:** 10.18632/oncotarget.4566

**Published:** 2015-06-22

**Authors:** Weiyang Chen, Jing-Dong J. Han

**Affiliations:** CAS Key Laboratory for Computational Biology, CAS-Max Planck Partner Institute for Computational Biology, Shanghai Institutes for Biological Sciences, Chinese Academy of Sciences, Shanghai, China

**Keywords:** Gerotarget

Aging has been a fascinating subject since ancient times. It is a process of gradual decline in the general function of an organism, manifested by age-dependent alteration and destabilization at the whole systems level [1]. It is the primary risk factor for many complex human diseases, including cardiovascular diseases, neural degenerative diseases, type 2 diabetes, and various cancers [2]. As aging is a system wide phenomenon, a systems biology perspective is needed to comprehensively and unbiasedly understand the regulation and intervention of aging. Below we summarize our attempts in this direction and then discuss the great potential of quantitative imaging in associating changes in molecular networks with the aging phenome.

First, by integrating interactome data with age-dependent gene expression data, we have constructed an active subnetwork for aging using protein-protein interaction networks by selecting interactions linking genes that are transcriptionally correlated or anti-correlated during the aging process [2, 3]. In both the human brain aging network and, the fruitfly aging network we have found transcriptionally anticorrelated gene clusters, corresponding to alternative cellular states, such as proliferation versus differentiation states or oxidative metabolic versus reductive metabolic states [3]. Surprisingly, the genes connecting transcriptionally anti-correlated modules are largely transcriptional and epigenetic regulators, including those regulating histone H3 lysine 27 trimethylation (H3K27me3).

We have further observed that the gene encoding the H3K27me3 demethylase UTX displayed a significant aging-dependent expression increase in the human brain, while its methytransferase Ezh2 decreases [4]. This aging-dependent increase also exists for the *C. elegans* ortholog *utx-1* gene, and reducing *utx-1* gene expression by RNAi delays the aging process and extends *C. elegans* lifespan by 30%. This lifespan extension requires a functional *daf-16* gene and acts through the IGF-1 pathway [4].

To identify common pathways affected by different dietary and energy interventions that modulate aging and lifespan, we obtained hepatic gene expression profiles of middle age mice on high-fat or low fat diet, with or without caloric restriction (CR), and with or without voluntary exercises. Pathway analysis showed that those pathways whose gene expression levels are associated with the mean lifespan across these different regimens are enriched for aging regulatory functions. This has allowed us to identify a set of known as well as novel pathways that are commonly targeted by different dietary and energy interventions and potentially mediate their effects on lifespan [5].

Most integrative analyses on aging, including our own works described above, have relied solely on molecular changes in the aging process. Moreover, to date, aging research has almost solely relied on mortality and lifespan as a gold standard for pro- or anti-aging determination. However, whether other phenotypes can replace mortality and lifespan as more robust aging and age evaluation benchmarks, in particular at the individual level; whether any individuals age faster or slower than the average; whether any parts of the body age faster or slower; and whether all aging-related molecular changes contribute to all individuals and all aging phenotypes equally or differentially, are questions that can only be effectively answered if the whole spectrum of the aging phenome be quantified and associated with molecular changes. The recent development of automated high throughput image acquisition technology and computational imaging informatics now allow for an unbiased and quantitative aging phenome to be automatically analyzed, and perhaps in the near future for this to take place in a high-throughput manner.

*C. elegans* is the most commonly used model organism for studying the molecular mechanisms of aging. We have, therefore, developed ‘WormFarm’, an integrated microfluidic device for culturing and monitoring *C. elegans* [6]. WormFarm allows for automated removal of progeny and efficient control of environmental conditions. In addition, we have developed computational image analysis algorithms to automatically analyze the video footage and quantify survival rate (by computationally counting live and dead worms) and other phenotypes, such as worm size, length, width, motility, shape and content density.

Using these quantified phenotypes, we have found long-lived *age-1(RNAi)* and *Y82E9BR.3 (RNAi)* worms significantly thinner and more mobile than their control groups. In contrast, the short-lived *sptf-3(RNAi)* worms is exactly the opposite. We have also examined the age dependent changes in worms expressing a mitochondrial-targeted GFP in muscle cells (*Pmyo-3::mito::GFP*) and have found a continuous decline in fluorescent intensity of GFP with age in worms. These phenotypic data demonstrate yet another significant advantage of the WormFarm culturing and imaging system, that is, automatic, simultaneous and quantitative analysis of multiple aging-related phenotypes [6].

With the knowledge that aging phenomes can be obtained by quantitative imaging analysis, we decided to use a 3D facial image capturing system to collect > 300 3D human facial images, well-distributed across 17–77 years of age. By analyzing these 3D facial images, we generated the first comprehensive atlas of the human facial aging phenome [7].

There are at least two types of phenotypes and/or biomarkers required to measure aging; one to measure the onset of aging and the other to reflect physiological age [2]. After aligning and transforming all vertices' values, we used the information of all 3D geometric vertices to extract aging-related facial changes. In this way, we have quantified facial morphological features and found that eye slopes, mouth width, nose width and the distance between the mouth and nose to be highly associated with age. In addition to predefined shapes and metrics, we have also uncovered other *de novo* aging related patterns. Most importantly, the 3D facial image data have allowed us to construct a robust age predictor, and based on the deviation between predicted and chronological age, to identify slow and fast agers. The classifications of fast and slow agers have also shown highly significant consistency with physiological indicators for health and age in participants' blood samples, suggesting that age predicted based on 3D facial morphology reliably reflects the biological age of an individual [7].

The aging “phenomics” now enabled by automatic and unbiased detection, quantification and analysis has emerged as a promising new dimension of information for the systems level analysis of aging. Integrating quantitative aging phenomics data with molecular and network changes will not only allow a more comprehensive understanding of aging at a higher level, but will also provide answers to questions that could not be addressed before, and thus generate new insights into the old problem of aging.
